# The Dual-Targeted Peptide Conjugated Probe for Depicting Residual Nasopharyngeal Carcinoma and Guiding Surgery

**DOI:** 10.3390/bios12090729

**Published:** 2022-09-05

**Authors:** Wenhui Huang, Zicong He, Xuekang Cai, Jingming Zhang, Wei Li, Kun Wang, Shuixing Zhang

**Affiliations:** 1 College of Medicine and Biological Information Engineering, Northeastern University, Shenyang 110167, China; 2 Medical Imaging Center, the First Affiliated Hospital, Jinan University, Guangzhou 510630, China; 3 CAS Key Laboratory of Molecular Imaging, The State Key Laboratory of Management and Control for Complex Systems, Institute of Automation, Chinese Academy of Sciences, Beijing 100190, China; 4 Department of Nuclear Medicine, Peking University First Hospital, Beijing 100034, China

**Keywords:** nasopharyngeal carcinoma, HER2, SR-BI, optoacoustic imaging, fluorescence molecular imaging, imaging-guided surgery

## Abstract

Detecting residual nasopharyngeal carcinoma (rNPC) can be difficult because of the coexistence of occult tumours and post-chemoradiation changes, which poses a challenge for both radiologists and surgeons using current imaging methods. Currently, molecular imaging that precisely targets and visualises particular biomarkers in tumours may exceed the specificity and sensitivity of traditional imaging techniques, providing the potential to distinguish tumours from non-neoplastic lesions. Here, we synthesised a HER2/SR-BI-targeted tracer to efficiently position NPC and guide surgery in living mice. This bispecific tracer contained the following two parts: IRDye 800 CW, as an imaging reagent for both optical and optoacoustic imaging, and a fusion peptide (FY-35), as the targeting reagent. Both in vitro and in vivo tests demonstrated that the tracer had higher accumulation and longer retention (up to 48 h) in tumours than a single-targeted probe, and realised sensitive detection of tumours with a minimum size of 3.9 mm. By visualising the vascular network via a customised handheld optoacoustic scan, our intraoperative fluorescence molecular imaging system provides accurate guidance for intraoperative tumour resection. Integrating the advantages of both optical and optoacoustic scanning in an intraoperative image-guided system, this method holds promise for depicting rNPC and guiding salvage surgery.

## 1. Introduction

Nasopharyngeal carcinoma (NPC) is an epithelial malignancy characterised by a distinct geographical distribution [1]. Up to 95% of patients diagnosed with NPCs are treated with radiotherapy [2]. Patients with early-stage NPC have encouraging survival rates, with the 5 year local control rates reaching 90%, while approximately 10% of patients with residual NPC (rNPC) demonstrate poor survival, and their 5 year local control rates are only 41% [3]. Recently, salvage surgery has been reported to provide effective tumour control, which is beneficial for achieving long-term survival in patients with local persistent NPC or rNPC [4]. However, intraoperative findings under endoscopic sinus surgery are inconclusive for delineating residual tumour margins, due to the presence of inflammatory tissue, fibrous hyperplasia, and oedema after radiotherapy [5]. Moreover, rNPC is not completely accessible preoperatively using conventional imaging techniques, with insufficient specificity [6]. Therefore, the accurate identification of rNPC has been a great challenge for both early detection and salvage surgery.

Recent advances in optical molecular imaging have the potential to exceed the specificity and sensitivity of conventional techniques for effective detection of small tumour foci both preoperatively and intraoperatively [7]. Optical molecular imaging allows for the visualisation of tumour-specific biomarkers in vivo via the smart utilisation of multiple molecular tracers [8]. When coupled with bispecific tracers, the specific interaction with two different targets might increase cellular uptake and simultaneously display longer retention in the tissues of interest, enabling longitudinal evaluations preoperatively and intraoperatively [9]. Several molecular biomarkers for NPC have been identified [10]. Among these, human epidermal growth factor receptor-1 (EGFR) has been verified as a traditional target for NPC. Numerous inhibitors, including cetuximab, gefitinib, and erlotinib, have been used in clinical trials [11,12]. Although these inhibitors have demonstrated clinical efficacy in some cases, several studies have not yielded favourable clinical benefits in rNPC, possibly owing to tumour heterogeneity. The unsatisfying results transfer researchers’ attentions to human epidermal growth factor receptor-2 (HER2), another member of the epidermal growth factor receptor family. The overexpression of HER2 was reported to range from 0% to 68% in head and neck tumours [13]. Furthermore, an analysis of immunohistochemistry in NPC specimens found HER2 positive in 33% of NPC patients [14]. These results indicate that HER2 might be a potential biomarker for NPC patients.

To achieve effective targeting, a single molecular target may not be sufficient. Recently, scavenger receptor class B type I (SR-BI), a receptor that selectively binds to high-density lipoprotein (HDL) and functions in lipoprotein metabolism, was reported to be overexpressed in all investigated NPC cell lines and 75% of NPC biopsies, demonstrating its promise in NPC imaging [15]. Thus, a dual-targeted peptide was synthesised, in which a HER2-specific sequence (LTVSPWYLTVSPWY) was linked to an SR-BI-specific sequence (FAEKFKEAVKDYFAKFWD) via a GSG linker, demonstrating its superior specificity in NPC xenograft tumours [16]. Therefore, targeting HER2 and SR-BI simultaneously might produce a cooperative targeting effect, which inspired us to adopt this peptide in our study.

Fluorescence-guided surgery is an optical imaging strategy that allows real-time guidance for detecting and resecting tumours intraoperatively with higher sensitivity than direct visual inspection and palpation [17]. Tumour-specific tracers provide a better tumour-to-background ratio (TBR) than nonspecific tracers, indicating potential for delineating tumour margins intraoperatively [18,19]. Generally, these tracers contain a near-infrared (NIR) fluorophore as an imaging reagent and a peptide or antibody as the targeting reagent. The clinical value of tumour-specific optical tracers in the operative setting has been emphasised in various types of tumours [20,21], although there is a lack of evaluation of its benefits in image-guided surgery for NPC. Thus, we intended to use the abovementioned bispecific tracer to explore its feasibility in preoperatively detecting NPC and intraoperatively delineating tumour margins in NPC mouse models. Additionally, intraoperative haemorrhage is a common complication in salvage surgery, suggesting that visualising the vascular network intraoperatively would reduce the injury induced by haemorrhage and improve the quality of life of patients with NPC. Considering that optoacoustic imaging has demonstrated a remarkable impact in vascular network imaging, its application in surgical guidance could compensate for the limitations of fluorescence molecular imaging (FMI) [22,23].

**Figure 1:**
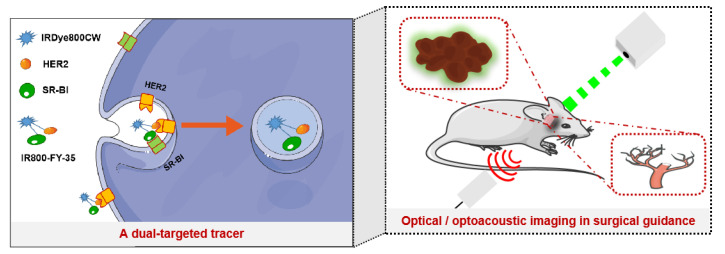
Schematic diagram of a dual-targeted tracer and optical/optoacoustic imaging in surgical guidance.

In this study, a bispecific peptide (FY-35) was labelled with IRDye 800CW (IR800-FY-35) and evaluated for its ex vivo and in vivo targeting efficacy (Figure 1). A single-target peptide (FL-35) was synthesised as a control. A customised hand-held optoacoustic scan system was introduced to visualise the tumour neovasculature. Surgical resection of NPC xenografts was performed under the guidance of our intraoperative FMI system.

## 2. Materials and Methods

### 2.1. Synthesis and Characterisation of Optical Tracers

The dual-targeted peptide FY-35 (FAEKFKEAVKDYFAKFWD, termed F; LTVSPWYLTVSPWY, termed Y) and a single-targeted peptide FL-35 (FAEKFKEAVKDYFAKFWD, termed F; SYTYSTPVWVPLWL, termed L) were obtained by solid-phase peptide synthesis in Apeptide Co., Ltd. (Shanghai, China). The synthesis cycle consisted of the following: (I) Fmoc cleavage: 20% piperidine in *N, N*-dimethylformamide (DMF), (II) DMF washings, (III) coupling, and (IV) DMF washings. The resin was treated with 2 mL dichloromethane (DCM) for 5 min three times and 2 mL DMF for 5 min three times before it was used. The resin was washed thoroughly before it was incubated with HBTU-preactivated amino acid, or commercially-available fluorophores (FITC/IRDye800 CW) activated ester. Cleavage from the solid support was performed with trifluoroacetic acid (TFA)/water/triisopropylsilane (95/2.5/2.5, vol/vol/vol) over 3 h at room temperature. All products were purified by high-performance liquid chromatography (HPLC) using a Gemini-NX 10μ C18 4.6 × 250 mm column (Phenomenex, Torrance, CA, USA) on a Shimadzu LC-2010A HPLC System (Kyoto, Japan). Mass spectroscopy was used to characterise the probes. The FITC-labelled probes were used for in vitro cell-binding assays.

### 2.2. Cell Culture

Human NPC cells 5-8F were transfected with firefly luciferase for in vivo imaging. Transfected cells (5-8F-fLuc) and non-transfected cells (5-8F) were cultured in high-glucose Dulbecco’s modified Eagle’s medium (DMEM), containing 10% foetal bovine serum, 1% penicillin, and streptomycin in a humidified atmosphere of 5% CO_2_ at 37 °C. The cells were harvested at 80% confluence and maintained in the exponential growth phase. Cell density was calculated using a haemocytometer and performed before any experiments.

### 2.3. Laser Confocal Microscopy

Confocal imaging was performed to explore the targeting efficiencies of FY-35 and FL-35. The 5-8F cells were cultured on confocal plates (500 μL of DMEM containing 5 × 10^2^ cells) for 24 h at 37 °C and then co-incubated with FITC-FY-35 or FITC-FL-35 (1.5 μM) for 1 h. The cells were then washed three times with phosphate-buffered saline (PBS) and fixed with 4% paraformaldehyde (PFA) for 8 min. Then, 4,6-diamidino-2-phenylindole and rhodamine B were used to stain the cellular nuclei and cytoskeleton, respectively. Immunofluorescence staining was performed using SR-BI and HER2 antibodies. The cells were fixed with 4% PFA for 8 min, permeabilised with 0.1% Triton™ X-100 for 15 min, and blocked with 2% bovine serum albumin (BSA) for 1 h at room temperature. The cells were then co-incubated with the SR-BI antibody (ab217318; 1:250 dilution in 0.1% BSA) or HER2 antibody (ab134182; 1:500 dilution in 0.1% BSA) overnight at 4 °C. After the standard washing process, the cells were co-incubated with rabbit anti-goat IgG H&L (ab150141) at a dilution of 1:1000 for 45 min at room temperature. All the above-mentioned staining processes were conducted away from light. Confocal images were acquired using a confocal laser scanning microscope (LSM-710, Carl Zeiss, Oberkochen, Germany). Imaging was performed using ZEN 2.3 lite (Zeiss, Oberkochen, Germany). Quantification of mean fluorescence intensity (MFI) was conducted using Image J 2.X (LOCI, University of Wisconsin, Madison, WI, USA).

### 2.4. Flow Cytometry Analysis

The 5-8F cells were cultured in 6-well plates (1.2 × 10^3^ cells/mL) for 24 h at 37 °C. When the cells reached the desired density, cells in the six plates were co-incubated with FITC labelled FY-35 or FL-35 at six concentrations (0, 1.5, 3, 6, 12, and 24 μM) for 1 h. Before flow cytometry analysis, the cells were washed three times with PBS, dissociated from the wells with trypsin–ethylenediaminetetraacetic acid (EDTA), and then resuspended in PBS. Cell counting was conducted using a flow cytometer (BD Accuri™ C6, BD Biosciences, Franklin Lakes, NJ, USA) with an excitation wavelength of 488 nm for FITC (525/30 emission filter). The data were analysed using FlowJo software (FlowJo 10.8, Ashland, OR, USA).

### 2.5. Animal Models

Animal experiments were performed in accordance with the approval of Animal Welfare and Ethics Committee of Jinan University (approval number: IACUC-20211210-03). Four-week-old BALB/c nude mice were purchased from the Beijing Vital River Laboratory Animal Technology Co., Ltd. (Beijing, China). Subcutaneous tumour models received a subcutaneous injection of 5-8F (5.0 × 10^6^ in 200 μL PBS) in the lower right flank. Experiments were started 10–14 days after tumour implantation, when the tumour diameter was 6–8 mm. Tumour volumes were measured using a Vernier calliper and calculated using the formula for a hemiellipsoid (volume = 0.5236 × length × width^2^), as this form best approximated the tumour shape [24]. For orthotopic tumour models, mice were anaesthetised using isoflurane with a precision vaporiser at 1% and injected with 25 μL of 1.1 × 10^5^ 5-8F-fLuc cells into the nasopharynx using a 28-gauge needle, as previously reported [25]. Mice were fed a specific pathogen-free unit, and animal surgery was performed in a sterile hood. Orthotopic tumours were visualised and tracked by bioluminescence imaging (BLI) using an IVIS^®^ Spectrum In Vivo Imaging System (PerkinElmer, Waltham, MA, USA). Specifically, mice received an intraperitoneal injection of 3 mg/mL luciferin bioluminescent substrate (Perkin Elmer, Waltham, MA, USA) 4 min before imaging.

### 2.6. Biodistribution and Tumour Selectivity of IR800-FY-35

Mice with subcutaneous 5-8F tumours were randomly divided into three groups (*n* = 3 per group). The three groups received intravenous (i.v.) injection with 12 μM FY-35 (dual-target dye), 12 μM FL-35 (single-target dye), and equimolar quantities of free dye, respectively. Fluorescence images were acquired pre-, 2 h, 6 h, 10 h, and 24 h post-injection. The mice in each group were euthanised. The heart, lung, liver, pancreas, spleen, kidney, intestine, and tumour tissues were collected for ex vivo imaging. The MFI was analysed to determine organ biodistribution and tumour selectivity in the three groups. The TBR was calculated by drawing regions of interest within the tumour region and ipsilateral auricle of each mouse.

### 2.7. Detecting Residual Tumours Using IR800-FY-35-Based Fluorescence Imaging

To identify the minimal size of detectable tumours, subcutaneous tumours of different sizes (1–7 mm; *n* = 8) were injected with 12 μM FY-35, and imaged at 24 h post-injection to measure MFI in the tumour region. A linear regression model was used to demonstrate a linear relationship between tumour volume and MFI. To confirm the feasibility of IR800-FY-35 for detecting residual tumours, mice with orthotopic tumours (*n* = 5) were intravenously injected with 12 μM IR800-FY-35. Fluorescent images at various time points (2, 4, 6, 8, 12, 24, and 48 h) were acquired with a 745-nm excitation wavelength and 800-nm filter using the IVIS^®^ Spectrum In Vivo Imaging System (PerkinElmer, Waltham, MA, USA). After 48 h post-injection, the mice were sacrificed. The orthotopic tumours and main organs were imaged ex vivo. To simulate salvage surgery in patients with NPC, we performed tumour resection on the sacrificed mice. Repeated imaging was performed to identify residual signals. The resected tumours were embedded in optimal cutting temperature (OCT) and stored at −80 °C. Serial sections were cut at a thickness of 10 μm for fluorescence imaging using an inverted microscope (Leica, Wetzlar, Germany), and 4 μm for haematoxylin–eosin (H&E) staining.

### 2.8. Optoacoustic Scanning

Three-dimensional (3D) volumetric optoacoustic imaging (MSOT Invision 128, iTheral Medical, München, Germany) was performed using a 384 ultrasound transducer with a centre frequency of 10 MHz organised in a hemispherical array with a radius of curvature of 4 cm. During the entire scanning, the mice were anaesthetised with an isoflurane-oxygen mixed gas (500 mL/min, Matrx VMR Small Animal Anesthesia Machine, Matrx, Dayton, OH, USA). Tumours were imaged at multiple wavelengths (710, 730, 740, 760, 770, 780, 790, 800, 810, and 850 nm). Raw data were reconstructed using the ViewMSOT software (iTheral Medical, München, Germany). We implemented spectral unmixing to resolve oxyhaemoglobin (HbO_2_) and IRDye800 CW, which are the two major contributors of the photoacoustic signal.

### 2.9. Imaging-Guided Surgery

Mice bearing subcutaneous NPC xenografts were IV injected with 12 μM IR800-FY-35, and surgery was performed at 24 h post-injection. The mice were anaesthetised, and the tumours were first subjected to optoacoustic imaging using a customised hand-held optoacoustic scanner to visualise tumour vessels. The tumours were then visualised and excised using the FMI intraoperative guiding system (DPM-III-01, Zhuhai Dipu Medical Technology Co., Ltd., Guangdong, China) with the advantage of convenient operation. The FMI intraoperative system is a dual-mode imager that combines the following two systems: NIR fluorescent imaging to detect targeted cancer cells, and visible-light reflectance imaging to observe the contours of the tissue itself. This system improves the image quality via feature point algorithms and allows rapid and precise imaging fusion for two cameras [26]. Surgically excised tissues were embedded in OCT, cut into serial sections, and subjected to H&E staining and confocal imaging.

### 2.10. Statistical Analysis

Statistical analysis was conducted using GraphPad Prism (9.0.0, GraphPad Software, San Diego, CA, USA). Data are reported as mean ± standard deviation. Comparative analysis was performed using an independent two-sample *t*-test or a chi-square test. Simple linear regression was used to estimate the relationship between two quantitative variables. Here, *p* < 0.05 was considered significant.

## 3. Results

### 3.1. Synthesis of Optical Tracers and Identification of Their Targeting Efficiency Ex Vivo

**Figure 2:**
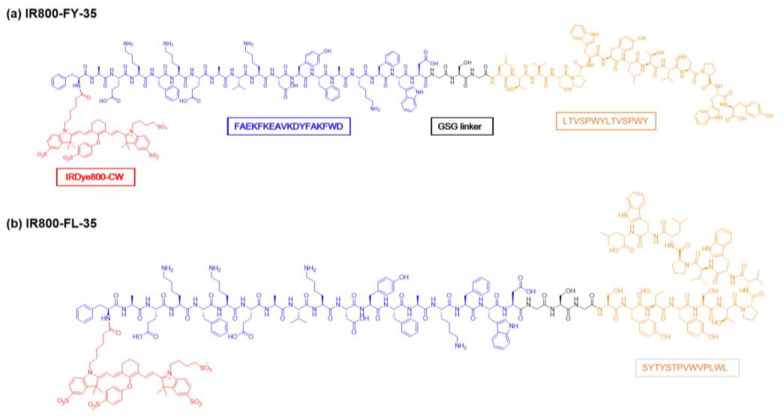
The chemical structure of IR800-FY-35 and IR800-FL-35. (**a**,**b**) The dual-targeted peptide FY-35 (sequence—FAEKFKEAVKDYFAKFWDGSGLTVSPWYLTVSPWY) and a single-targeted peptide FL-35 (sequence—FAEKFKEAVKDYFAKFWDGSGSYTYSTPVWVPLWL) were obtained by solid-phase peptide synthesis and labelled with IRDye 800CW.

**Figure 3:**
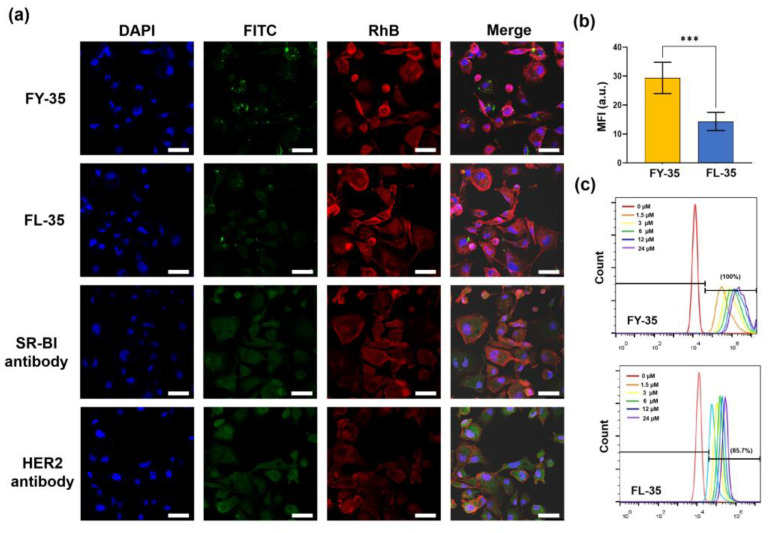
The cellular uptake in 5-8F cells of two probes was visualised and analysed using confocal imaging and flow cytometry analysis. (**a**) Representative confocal images demonstrating the selective uptake of FITC labelled dual-targeted ligand (FY-35) and single-targeted ligand (FL-35) in 5-8F cells. The fluorescent signal was shown in the cytomembrane and cytoplasm, which was consistent with the results of HER2 or SR-BI immunofluorescence staining. Scale bar = 20 μm. (**b**) Quantitative analysis of mean fluorescent intensity (MFI) confirming the significant difference in the cellular uptake of two probes. (**c**) Quantitative analysis of two probes in selective binding with 5-8F cells by flow cytometry. Abbreviations are as follows: DAPI, 4′,6-diamidino-2-phenylindole; RhB, Rhodamine B dye; FITC, fluorescein isothiocyanate. Here, ***, *p* < 0.001.

Here, FY-35 was a fusion peptide targeting both HER2 and SR-BI for our imaging studies, while FL-35 was designed as a single-target control, only binding with SR-BI. The chemical structure of IRDye 800 CW-labelled FY-35 and FL-35 were shown in Figure 2. The FITC-labelled FY-35 and FL-35 were synthesised for ex vivo tests (Figure S1, Supplementary Materials). The mass spectra were presented in Figures S2 and S3, and the purity of these products was about 98% (Figures S4 and S5). The targeting efficiency was first identified by confocal imaging and flow cytometry analyses in vitro. Based on effective receptor-mediated endocytosis, probe uptake within the two groups was qualitatively and quantitatively analysed (Figure 3a,b). As indicated in Figure 3a, bright green fluorescence was observed in the cytomembrane and cytoplasm of each group, indicating that the two tracers were easily internalised by cells. The cellular uptake of FITC-FY-35 by 5-8F cells was significantly higher than that of FITC-FL-35 (*p* = 0.0001). Immunofluorescence staining demonstrated stable co-expression of SR-BI and HER2 in 5-8F cells, providing the basis for effective targeting of the two tracers. The flow cytometry results further validated the findings of confocal imaging (Figure 3c). With increasing co-incubated concentrations of FITC-FY-35 and FITC-FL-35, the fluorescent peaks shifted to the right, indicating that the number of probe-binding cells was positively correlated with the concentration. Although the two tracers demonstrated selective binding with 5-8F cells, the ratio of marked cells in the FITC-FY-35 group (100%) was higher than that in the FITC-FL-35 group (84.7%) at the first concentration (1.5 μM), demonstrating the additional targeting capacity of FY-35.

### 3.2. Biodistribution and Tumour Selectivity of IR800-FY-35

**Figure 4:**
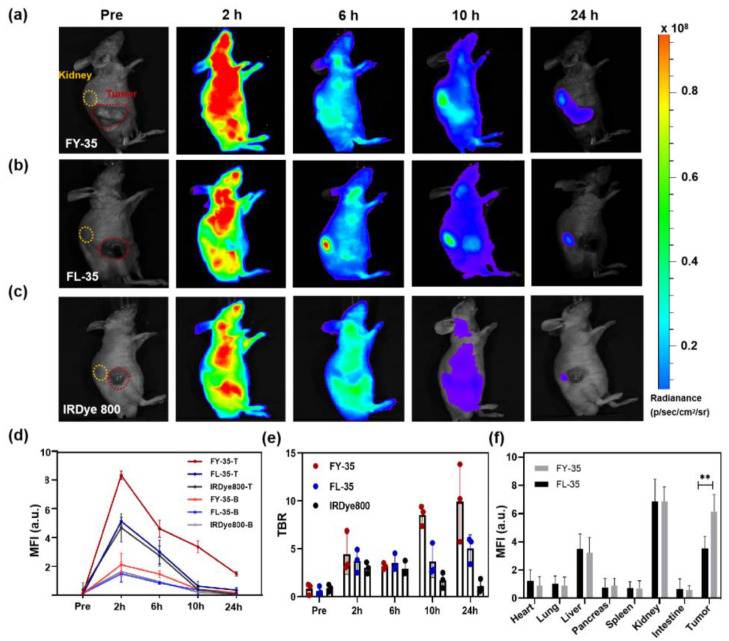
IR800-FY-35 effectively targeted 5-8F tumours and offered better performance than IR800-FL-35 on whole-body fluorescence imaging. (**a**–**c**) Serial images of representative mice bearing 5-8F tumours in three groups. Equimolar quantities of IR800-FY-35, IR800-FL-35, or IRDye800 CW (five mice per group) were intravenously administrated to tumour-bearing mice and imaged pre, 2, 6, 10 and 24 h post-injection. The contour of tumours and the right kidney were marked by the red dashed line and orange dashed line, respectively. (**d**) The mean fluorescence intensity (MFI) from tumour regions in three groups at various timepoints. (**e**) The tumour-to-background ratio (TBR) collected on the whole time points (Pre, 2 h, 6 h, 10 h, 24 h) from three groups. (**f**) Ex vivo MFI analysis for the resected organs and tumours in IR800-FY-35 and IR800-FL-35 at 24 h post-injection. Here, **, *p* < 0.01.

To further explore in vivo targeting efficiency, a biodistribution test was performed using a subcutaneous NPC mouse model. At 2 weeks following tumour inoculation, the tumour-bearing mice were randomised into three groups (*n* = 5 per group) with equal average tumour burden (Figure 4a–c). The mice were IV administered IR800-FY-35, IR800-FL-35, or free dye. Similarly, both targeted tracers revealed apparent tumour selectivity at 6 h and 10 h post injection, indicating that SR-BI and HER2 might be suitable imaging markers for NPC. Significantly, the higher signal intensity within the tumour region (3.575 ± 2.953) in the IR800-FY-35 group was demonstrated in comparison with the IR800-FL-35 group (1.835 ± 2.178) or free dye group (1.65 ± 1.923) (Figure 4d,e). The maximum TBR was 13.83, which was 2.3-fold higher than that of IR800-FL-35 (maximum TBR = 5.99). Except for the superior optical contrast, the longer retention of IR800-FY-35 in 5-8F tumours was observed at 24 h after injection, demonstrating the potential for applying this tracer in imaging-guided surgery, since preoperative preparation in clinical practice generally requires more time. Ex vivo fluorescence imaging of the organs and tumours resected from the two groups of mice demonstrated that both tracers mainly accumulated in the kidney, liver, and tumours (Figure S6). Quantitative analysis of MFI revealed a significant difference in tumour uptake between the two groups (*p* = 0.0079; Figure 3f). Altogether, IR800-FY-35 demonstrated a higher binding affinity and longer retention in NPC than IR800-FL-35.

### 3.3. Detecting Minimal Residual NPC Using IR800-FY-35

**Figure 5:**
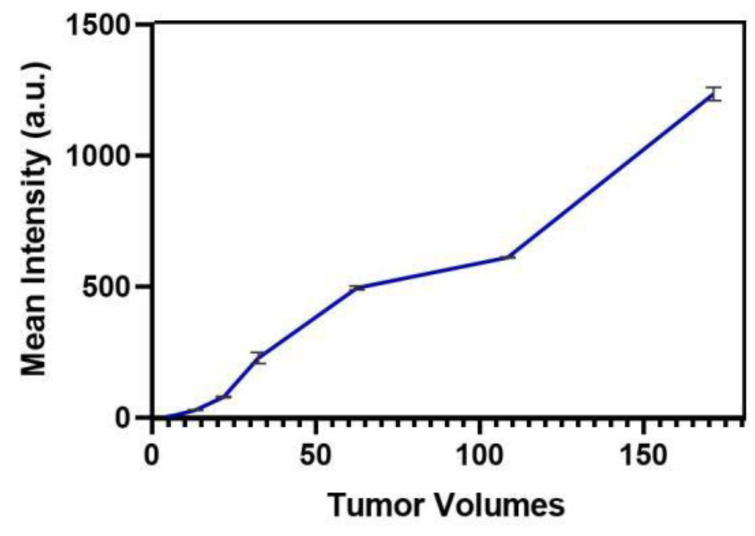
The linear relationship between two variables.

**Figure 6:**
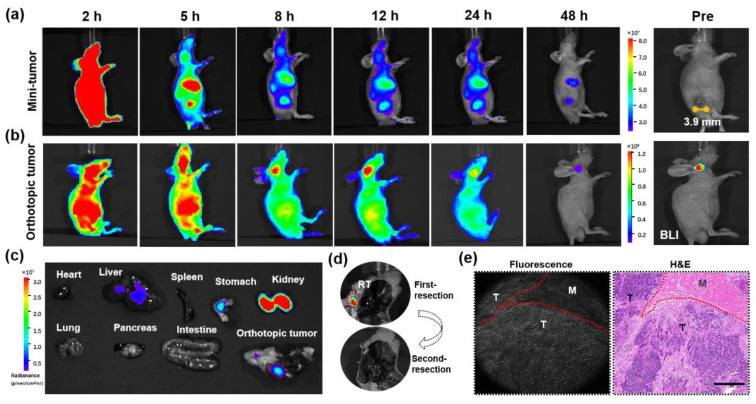
IR800-FY-35 identified residual tumours and helped the surgical resection of the infiltrative tumour in orthotopic model under fluorescence guidance. (**a**) Serial images from mice bearing subcutaneous tumours with the minimal size of 3.9 mm. (**b**) Pre-operative detection of orthotopic tumours on serial images after IV injection of IR800-FY-35. The bioluminescent image (BLI) confirmed the tumour location. (**c**) Ex vivo fluorescent image of organs and the orthotopic tumour. The solid tumour infiltrating right oropharynx did simulate human NPC. (**d**) Post-operative images after the first and second surgery. The residual signal at first resection was absent after the second resection. RT represented the suspiciously residual tumour. (**e**) Fluorescence slice and corresponding H&E staining of the resected tissue. The residual tumour region (T) showed a higher signal than adjacent muscle tissue (M). Scale bar = 500 μm. Abbreviations are as follows: IV, intravenous; NPC, nasopharyngeal carcinoma; H&E, haematoxylin–eosin.

To confirm the feasibility of IR800-FY-35 in identifying residual tumours, we first tested the minimal size of tumours that could be detected in subcutaneous tumours. Mice bearing 5-8F tumours (*n* = 8) of different sizes (1–7 mm) were injected with equivalent IR800-FY-35 and imaged at several time points. The MFI in the tumour region at the 24 h time point was recorded in each mouse, and then a linear regression model was performed to exhibit a linear relationship between the tumour volumes and MFI. We observed a linear relationship between these two variables (Y = 7.178 × X-41.31) (Figure 5). The MFI increased remarkably when the tumour volume reached 32 mm^3^, indicating that the minimum size of detectable tumours was 3.9 mm (Figure 6a).

To further evaluate the feasibility of IR800-FY-35 in detecting residual tumours, an orthotopic mouse model (*n* = 5) was constructed by injecting 5-8F-fLuc cells into the nasopharynx and experimented when the bioluminescent signal reached 10^8^. Considering that the parotid gland presented apparent probe uptake (Figure 4a–c), the delayed imaging time points might have avoided troublesome signal interference. Thus, mice with orthotopic tumours were imaged at 2, 5, 8, 12, 24, and 48 h post-injection. Similarly, superior optical contrast was observed in the tumour region, which was consistent with that in the subcutaneous tumour, but surprisingly persistent until 48 h post-injection (Figure 6b). The mice were then euthanised and exposed to the tumours and the main organs (Figure 6c). To simulate salvage surgery, we performed tumour resection ex vivo. Following the first white-light resection and removal of the macroscopic tumour, the residual signal was adjacent to the tumour bed, as shown in Figure 6d, indicating a suspicious tumour that was not easily detectable under white light. The presence of cancer cells was confirmed by H&E staining. Cancer cells exhibited strong NIR signals in fluorescence microscopy imaging, whereas the muscle had weak signals on the same slice (Figure 6e). These results verified the feasibility of IR800-FY-35 in detecting minimal residual NPC.

### 3.4. Photoacoustic and Fluorescence Imaging-Guided Resection

**Figure 7:**
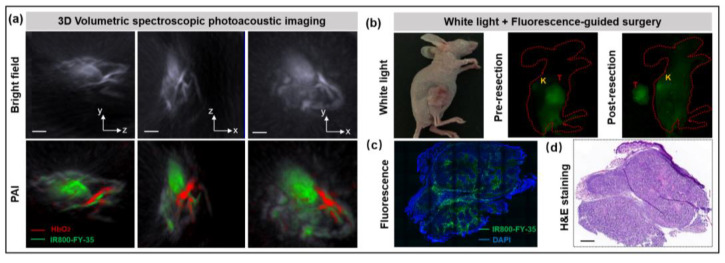
The IR800-FY-35-based intraoperative FMI system successfully guided tumour resection. (**a**) In vivo 3D volumetric spectroscopic photoacoustic imaging for preoperatively mapping tumour (green) and tissue micro-vessels (red) via spectral unmixing of IR800-FY-35 and HbO_2_. Scale bar = 5 mm. (**b**) Intraoperative images of 5-8F tumour bearing mice under the white light and the fluorescence channel before and after surgery. (**c**) Confocal imaging representing the strong IRDye800 CW signal in tumour slice. The nuclei were stained with 4′,6-diamidino-2-phenylindole (DAPI). (**d**) Corresponding H&E staining from the same cross-section. Scale bar = 1 mm. Abbreviations are as follows: FMI, fluorescence molecular imaging; HbO_2_, oxyhaemoglobin; PAI, photoacoustic imaging; H&E, haematoxylin&eosin.

The common complications of salvage surgery are intraoperative haemorrhage and inadvertent damage to the carotid vessel; intraoperative tumour vascular and adjacent artery imaging could effectively reduce surgical complications. To avoid severe bleeding in orthotopic mouse models due to tumour infiltration into adjacent muscle tissues, the subcutaneous tumour model (*n* = 5) was IV injected with IR800-FY-35, and image-guided resection was conducted using both photoacoustic and fluorescence imaging. The 3D volumetric optoacoustic imaging was performed to clearly visualise the tumour vessels at 24 h after administration (Figure 7a). Subsequently, a surgeon attempted to resect all tumours completely under white light, while carefully ligating the tumour vessels to reduce bleeding during the procedure. The resected tumour and postoperative tumour bed are presented in Figure 7b. Confocal imaging of tumour slices revealed accurate microscopic co-localisation of tumour cells and the IRDy800 signal (Figure 7c,d). Based on these imaging data, the dual-targeted tracer could enhance the ability of surgeons to accurately conduct tumour resection by integrating fluorescence with optoacoustic imaging to guide surgery.

## 4. Discussion

Here, we conjugated IRDy800CW to produce a fusion peptide (FY-35) that targeted HER2/SR-BI, and confirmed its superior targeting efficiency and longer retention in NPCs, with the contrast being improved 2.3-fold at 24 h post-injection and the retention time extended up to 48 h, when compared to a single-targeted tracer (IR800-FL-35). These results reveal the potential for IR800-FY-35 to be utilised for preoperative detection and to guide intraoperative resection of NPCs. Although it is not technically feasible to perform salvage surgery in live mice with orthotopic NPC, resection surgery on the executed mice with orthotopic tumours simulated the difficulty of residual tumour visualisation under white light and identified the feasibility of IR800-FY-35 in detecting minimal residual tumours. Moreover, with the good performance of vascular network mapping by 3D volumetric optoacoustic imaging, surgical excision of subcutaneous NPC tumours was successfully achieved with the guidance of our intraoperative FMI system.

Despite advancements in NPC therapy, approximately 10% of patients develop residual or recurrent disease; the genomic landscape of rNPC is complex, with enriched mutations leading to enhanced tumour heterogeneity [27]. The dual-targeting strategy with two binding receptors copes with heterogeneity and allows for improved uptake in the region of interest for specific imaging or targeted therapy [9]. Here, the HER2/SR-BI targeting tracer in our study indicated that the addition of another binding monomer had advantageous effects on cellular internalisation and tumour uptake (Figure 3 and Figure 4). Both in vitro and in vivo tests of FY-35 revealed significantly improved binding affinity with cancer cells, which was consistent with previous results [15].

Except for superior targeting efficiency, longer retention in the tumour region can provide a one to two day window that offers flexibility in scheduling surgical procedures. There are many platforms for tumour-targeted fluorescent probes [28]. Besides a peptide probe in our study, small molecules and antibodies are available in tumour-targeting platforms for NPC. Antibodies, the most widely used platform, have effective accumulation and longer retention in tumours, but suffer from pharmacokinetic limitations, including slow tissue clearance (normally 2–5 days or longer) and non-specific organ uptake [7]. Small molecules or peptide probes show rapid diffusion into the circulation blood stream, with the advantage of low cost and minor side effects and, thus, have promise in clinical settings [29]. However, the short retention time in tumours is a disadvantage of peptide probes when utilised in fluorescence-guided surgery [30]. Here, the dual-targeted probe in our study surprisingly makes up for the disadvantage, with the longest retention time reaching to 48 h. There may be two explanations for the durable tumour accumulation. First, doubling the binding monomer can double the molecular weight of the targeted tracer, leading to a long circulatory half-life and slow clearance. Second, the HDL receptor SR-BI is critical for cholesterol haemostasis, and the ApoA-I mimetic peptide (FAEKFKEAVKDYFAKFWD) could function as HDL and possibly increase the efficiency of receptor-mediated endocytosis, contributing to its remarkable accumulation in tumours. Previously, several studies underscored the use of reconstituted HDLs as target-specific nanoparticles for drug carriers to tumours, and highlighted that these peptides can serve as safe targets for a number of cancers [31,32]. In addition to the good performance of the reported peptide, IRDye 800CW was selected because of its safety and preliminary success in several clinical studies [19,33]. These studies have reported its low autofluorescence and good quantum yield in receptor-targeted fluorescence-guided surgery and, most importantly, its compatibility with both the fluorescence-guiding system and optoacoustic scanning system. These findings encouraged us to examine the capacity of the dual-targeted tracer to detect residual tumours and delineate tumour margins in optical and optoacoustic-guided surgery.

The imaging strategy used in our study has the potential for translation into clinical practice. First, both fluorescent and optoacoustic imaging systems were approved by the US Food and Drug Administration. Second, a fibre-delivered optoacoustic guide was previously reported, which mimicked the traditional guide wire and allowed intraoperative placement into the tumour mass to visualise the tumour with sub-millimetre variance [34]. Considering the compatibility of the flexible fibre optoacoustic guide with any conventional endoscope and the advancement of hand-held optoacoustic scanning systems [23,35], it can be incorporated into endoscopic nasopharyngectomy. Optoacoustic imaging possesses the advantages of both optical and ultrasonography that offers a superior vertical resolution at a depth of 2–4 cm and excellent vascular network mapping [36]. Considering that fluorescence-guided endoscopy has been widely reported [37,38], the complementation of optoacoustic imaging would compensate for the limitations of optical-guided surgery [39,40]. Altogether, a possible clinically related image-guiding strategy for endoscopic surgery in rNPC should be as follows. Optoacoustic imaging can be used for localisation with tissue microvasculature and tumour infiltration depth at the microscopic scale. Then, NIR imaging can be used to depict tumour boundaries on a macroscopic scale.

There are several limitations in our study. Firstly, we only selected the peptide FL-35 that had the same molecular weight as FY-35, but only targeted SR-BI as a single-target control. To evaluate both, two separate peptides as control would be more persuasive. Secondly, while we successfully performed tumour resection under fluorescent and optoacoustic imaging guidance, there was a lack of analysis concerning the volume of intraoperative bleeding, which would be worthy of testing in our future study.

## 5. Conclusions

Our data demonstrated the power of the dual-targeted tracer for optoacoustic and fluorescence-guided surgery. Utilisation of this method enables the detection of residual tumours and tumour delineation in NPC in vivo, providing a basis for image-guided surgery. Combining the advantages of optical imaging (i.e., wide field-of-view) with optoacoustic scanning (i.e., high-resolution and vascular network mapping), this method holds promise in guiding salvage surgery for rNPC, which might reduce reoperation rates and shorten surgery time.

## Data Availability

Not applicable.

## Supplementary Materials

The following supporting information can be downloaded at: https://www.mdpi.com/article/10.3390/bios12090729/s1, Figure S1: The chemical structure of FITC-FY-35 and FITC-FL-35; Figure S2: Mass spectra of FITC-FY-35; Figure S3: Mass spectra of FITC-FL-35; Figure S4: HPLC for FITC-FY-35; Figure S5: HPLC for FITC-FL-35; Figure S6: Ex vivo fluorescent imaging for the resected organs and tumours in two groups.
