# Supplementary material for: The Dual-Targeted Peptide Conjugated Probe for Depicting Residual Nasopharyngeal Carcinoma and Guiding Surgery
Source: Biosensors (Basel). 2022 Sep 5;12(9):729. doi: 10.3390/bios12090729 (PMC9496553; doi:10.3390/bios12090729)
Supplement: Supplementary file 1 [file biosensors-12-00729-s001.zip › biosensors-1869026-supplementary.pdf]

biosensors

MDPI

Supplementary Materials

The Dual-Targeted Peptide Conjugated Probe for Depicting Residual Nasopharyngeal Carcinoma and Guiding Surgery

Wenhui Huang [1,2,3], Zicong He [2], Xuekang Cai [4], Jingming Zhang [4], Wei Li [2,3], Kun Wang [3,*] and Shuixing Zhang [2,*]

1 College of Medicine and Biological Information Engineering, Northeastern University, Shenyang 110167, China
2 Medical Imaging Center, the First Affiliated Hospital, Jinan University, Guangzhou 510630, China
3 CAS Key Laboratory of Molecular Imaging, the State Key Laboratory of Management and Control for Complex Systems, Institute of Automation, Chinese Academy of Sciences, Beijing 100190, China
4 Department of Nuclear Medicine, Peking University First Hospital, Beijing 100034, China
* Correspondence: kun.wang@ia.ac.cn (K.W.); zsx7515@jnu.edu.cn (S.Z.); Tel.: +86-13544597585 (S.Z.); +86-18612965656 (K.W.)

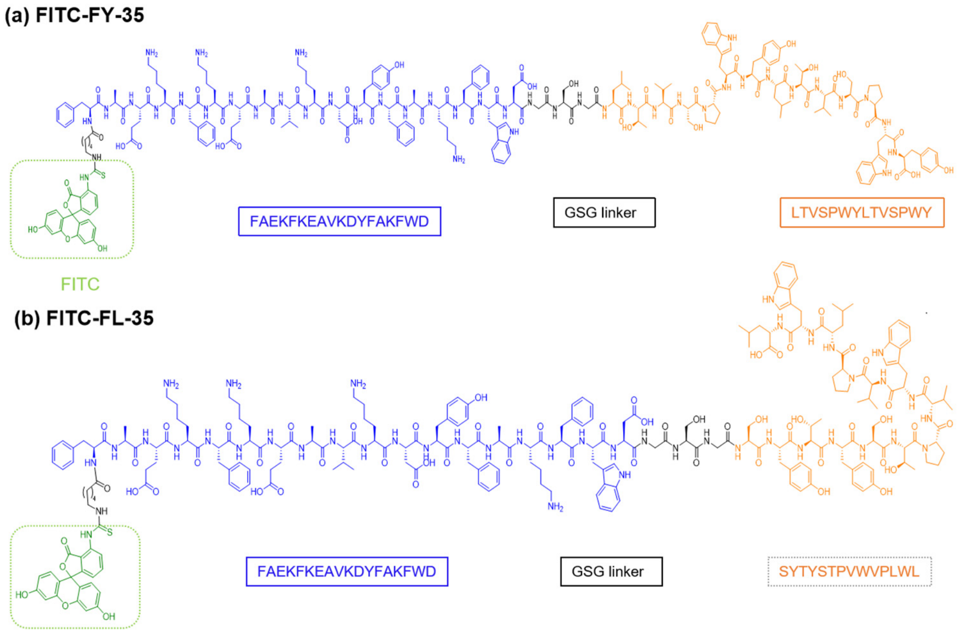

Figure S1. The chemical structure of FITC-FY-35 and FITC-FL-35.

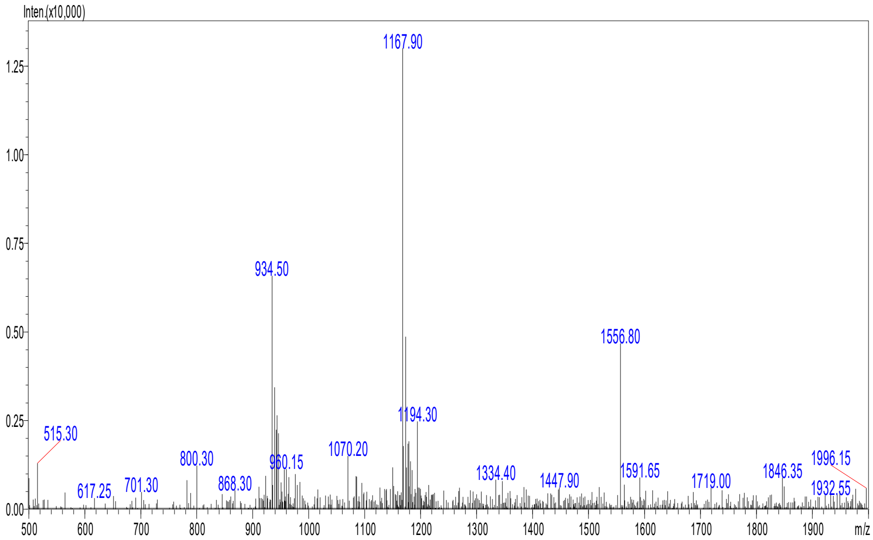

Figure S2. Mass spectra of FITC-FY-35.

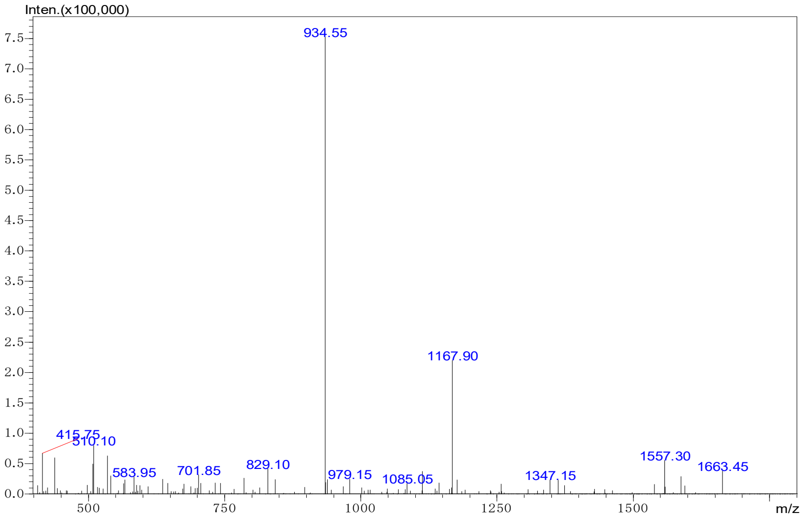

Figure S3. Mass spectra of FITC-FL-35.

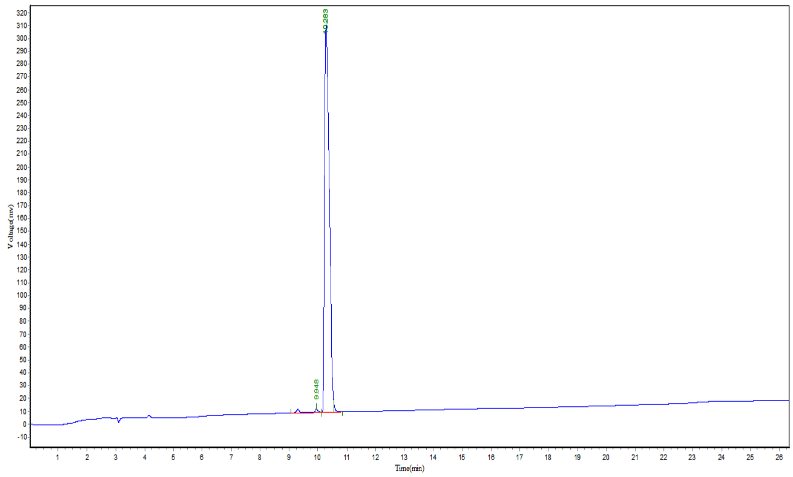

Figure S4. HPLC for FITC-FY-35.

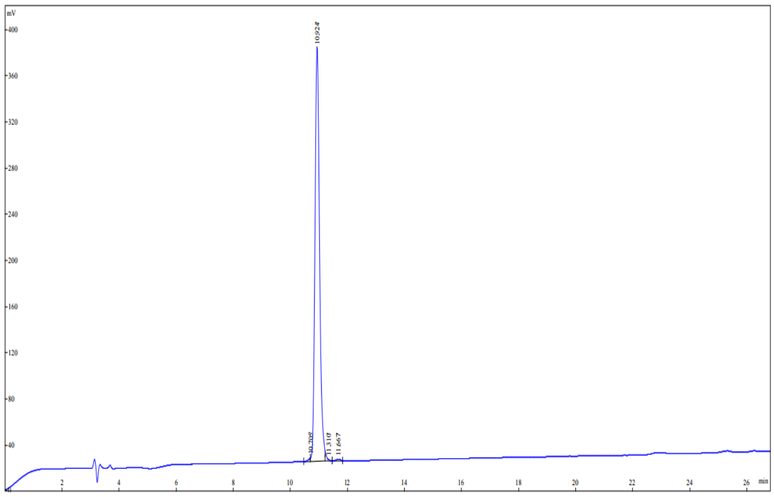

Figure S5. HPLC for FITC-FL-35.

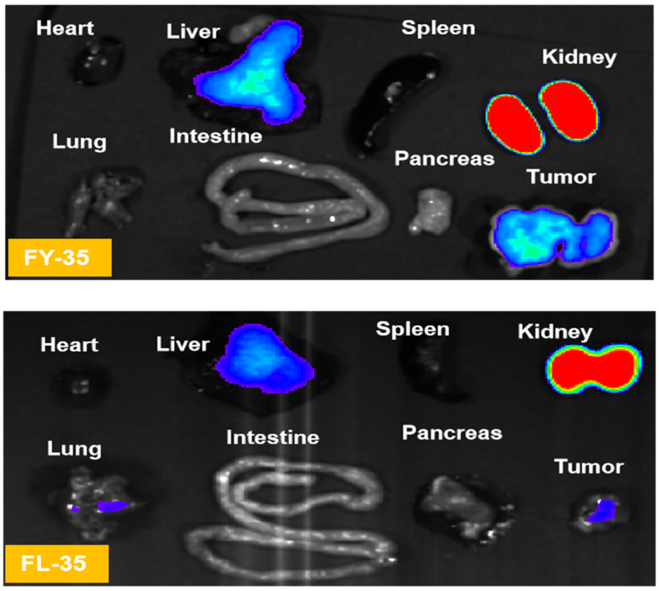

Figure S6. *Ex vivo* fluorescent imaging for the resected organs and tumours in two groups.
